# Are Structural Changes in Polish Rural Areas Fostering Leisure-Time Physical Activity?

**DOI:** 10.3390/ijerph14040372

**Published:** 2017-04-01

**Authors:** Elżbieta Biernat, Piotr Bartkiewicz, Sonia Buchholtz

**Affiliations:** 1 Collegium of World Economy, Warsaw School of Economics, al. Niepodległości 162, 02-554 Warszawa, Poland; 2 Collegium of Economic Analysis, Warsaw School of Economics, al. Niepodległości 162, 02-554 Warszawa, Poland; pb34168@sgh.waw.pl (P.B.); sb43146@sgh.waw.pl (S.B.)

**Keywords:** leisure-time physical activity, farmers, rural areas, Poland, propensity score matching, Heckman selection modelling

## Abstract

*Background:* In this study, we analyze the determinants of leisure-time physical activity (LTPA) of farmers and non-farmers living in rural areas. *Methods:* We use statistical analysis to describe urban and rural populations, as well as econometric techniques (Heckman regressions and propensity score matching) to assess the role of rural lifestyle in physical activity. *Results:* World Health Organization (WHO) pro-health PA (physical activity) recommendations are not met by 66% of farmers and 49% of other dwellers in rural areas. Approximately two thirds of them are completely inactive. Farmers enjoy vigorous PA (VPA), cycling and recreational walking less than their non-farming counterparts and are 46% less likely to be active than them; however the difference disappears when they take up an activity. The amount of PA is negatively correlated with age, but tends to increase for older people compared to those in middle age. Women are 6%–7% less active than men, yet the odds of being active at all are higher for women than for men. Household size is negatively correlated with LTPA. *Conclusion:* Considering the structural changes, rural area dwellers, especially farmers, require public intervention aimed at increasing their awareness of the advantages of LTPA.

## 1. Introduction

Approximately two in five Polish citizens live in rural areas [1], which makes the rate of urbanization lower than in most European Union (EU) countries. As a matter of fact, rural dwellers are in a relatively worse position than their urban counterparts. According to data from the Central Statistical Office of Poland (CSO), they live shorter lives [2], have a poorer educational background [3] and enjoy systematically lower earnings [4]. Not surprisingly, young people are leaving the countryside [5]. In order to counteract these trends, rural areas are diversifying away from farming, however, this process is only moderately successful [6]. Even though the role of agriculture has been significantly decreasing since the fall of the Iron Curtain, it still involves many resources, and makes a very small contribution to gross output. Small farms limit labor productivity [7] although the capital-labor ratio has experienced significant improvement during the last 15 years, mainly due to EU subsidies [8].

Work in agriculture has undergone huge changes due to technological progress. In particular, many tasks no longer require physical effort, as they are carried out by machines. As a consequence, contemporary farming is becoming more like office work, characterized by its sedentary nature [9]. However, it is still highly seasonal, involves static effort or engages small groups of muscles in a repetitive way [10], which does not necessarily favor physical fitness [11]. The existing literature indicates that large-area farmers rarely compensate their hard, occupational work with leisure-time physical activity (LTPA) (see [12] for China and [13,14] for Australia), but recent evidence for small-area farmers is rather scarce [15,16,17]. Moreover, the lives of farmers and inhabitants of rural areas often overlap. This creates a risk of over-interpretation [18], as both groups represent sedentary lifestyles and are vulnerable to insufficient physical activity [12,19]. Therefore, one should establish whether the key factor in inactivity is rural dwelling, simply being a farmer, or their correlates. Regardless of these considerations, physical inactivity and a sedentary lifestyle are proven to cause a wide range of non-communicable diseases (NCDs) affecting circulatory and locomotive systems (from cardiovascular diseases to musculoskeletal symptoms through diabetes and hypertension). Their primary causes are being overweight or obese and a lack of leisure-time physical effort [17].

Polish rural areas are experiencing four trends: diversification away from farming, expanding the average farming area, mechanization of the agricultural production, and population ageing. In the nearest future, the number of people employed in agriculture should decline. From a theoretical standpoint, the impact of these tendencies on individual physical activity is mixed—the first should improve their activity-related habits, but the last two carry the risk of increases in insufficient effort. As physical activity declines when individuals age and gradually lose their physical fitness, cohort effects (i.e., younger generations are more active than their older colleagues at the same point in their lives) could mitigate that effect. The overall direction of change is thus unknown. In this article, we analyze how the abovementioned structural changes of rural areas are affecting the physical activity of the rural dwellers and, particularly, farmers. We attempt to answer three questions: (1) to what extent does the Polish pattern of inactivity apply to Polish farmers and rural dwellers? (2) does the status of farmer or rural inhabitant *per se* determine inactivity, or are there any covariates affecting LTPA? and (3) will structural changes in Polish rural areas raise LTPA? 

## 2. Materials and Methods

The analysis was conducted on the basis of a dataset from a LTPA survey in Poland, commissioned by the Polish Ministry of Sport and Tourism. Three cross-sectional waves of the survey conducted between 2014 and 2015 described socio-demographic characteristics of individuals, representative of the Polish population, and various aspects of physical activity: recreational walking, transportation walking, cycling, moderate physical activity (MPA) and vigorous physical activity (VPA). The respondents were asked about the frequency and duration of their LTPA in the week preceding the survey, provided that it was a regular week. In order to avoid a seasonality, and to average weather conditions, subsequent waves were conducted in March and November.

Declared activity was converted to physical effort (in MET-minutes) on the basis of the World Health Organization (WHO) framework [20]. According to its guidelines, in order to maintain good health, one should provide at least 601 MET-min/week of physical effort, which equals approximately 180 min of walking, 100 min of cycling or 75 min of VPA in at least 10 min periods (For a technical description of MET-min calculations see [21]). Individuals failing to provide this effort were called low physically active. Those, whose weekly LTPA oscillated between 601 and 1500 MET-min, belonged to the moderately physically active, while those exceeding 1500 MET-min per week—highly physically active. This division was crucial for further analysis. The survey followed the rules of the short version of the International Physical Activity Questionnaire (IPAQ), enabling cross-country comparisons. Compared to the long version, it omits occupational and transportation activity. However, as 10 min periods apply, and respondents are given the examples of LTPA, the risk that LTPA and occupational PA will be confused, is minimized [22].

In order to gain a sample large enough for an in-depth analysis, we merged the datasets from consecutive waves. This was possible as the respondents in each wave answered similar questions and samples were cross-sectional. The combined sample covered 3051 respondents, of whom we distinguished two subsamples: rural dwellers (*n* = 1172) and farmers (*n* = 233), for the sake of further analysis. The sample contained 210 rural farmers, 23 urban farmers, and 962 rural inhabitants working outside of agriculture. The details of subsamples are presented in Table 1.

There are several ways to measure the association between physical activity and being a farmer (Analogous analysis can be drawn for inhabitants of rural areas). One approach is to make use of quasi-experimental econometric methods and compare farmers to their closest *twins* among non-farmers. This category of methods allows for easy comparison of the physical effort while controlling for socioeconomic covariates (age, household size, socioeconomic status (SES), etc.) in a way that is closest to a truly experimental setting (which was beyond the scope of this study). The matching procedure took place between the observations from the treatment group (i.e., being a farmer) and the control group (i.e., working outside agriculture). In order to assess the impact of being a farmer on physical activity, we used propensity score matching (PSM) with one-to-one matching. The propensity score, a synthetic approximation of the difference between the treatment and control groups, was calculated with a logistic regression. While the range of possible choices regarding both the model used for calculating propensity scores, and the method of matching units from treatment and control groups was quite wide, we opted for one of the most common ones—nearest neighbor matching (Nearest neighbor matching for every unit from the treatment group the algorithm searches for the single most similar unit from the control group). Robustness checks confirmed suitability of that choice.

The second approach we utilized was to analyze the impact of various demographic and socioeconomic factors on the amount of physical activity and, ultimately, to extract the influence of the place of residence and profession on the amount of physical activity an individual enjoys. We hypothesized that the choice regarding physical activity is a two-stage process and involves deciding on whether to take up physical activity at all (no activity vs. positive amount of it) and on the form and amount of the activity. As a result, the distribution of the amount of physical activity is a peculiar one: there is a large number of zeros (23% of respondents report no physical activity at all) and a fairly symmetrical, unimodal distribution outside of zero. Applying simple ordinary least squares regressions to this dataset would bias all the coefficients and overstate the influence various factors have on the level of physical activity [23]. To tackle this problem, we decided to use a Heckman selection model which explicitly assumes a two-stage process generating the relevant variable: a selection equation and an outcome equation [24,25]. This approach allows for calibrating both equations separately, i.e., estimating the impact of the choice to be active, and different factors on the level of physical activity. We chose the natural logarithm of physical activity as the dependent variable. Aside from the ease of interpretation (The parameters of such model have the interpretation of an effect size, i.e., the percentage change in the dependent variable (amount of physical effort) associated with an incremental change in each regressor) its advantage lies in reducing the problem of heteroscedasticity present in cross-sectional data of similar characteristics, due to massive reductions in the variance of the dependent variable and, by extension, the residuals. In all instances, model specification was chosen on the basis of information criteria.

The PSM and Heckman regression modelling are supplemented with descriptive statistics, including the decomposition of treatment effect into 5 types of physical activity. Throughout the paper, both in density estimates and in Heckman selection regressions, we calculate *p* = ln (1 + *P*) as our preferred measure of physical activity, where *P* is the weekly amount of physical activity in MET-min. The reasons are purely pragmatic: a more practical interpretation of results, clarity and the opportunity to avoid erroneous ln (0) for respondents who report zero physical activity. Quantitative analysis was conducted with STATA 14 software (StataCorp LP, Lakeway Drive, TX, USA).

## 3. Results

### 3.1. Prevalence of Leisure-Time Physical Activity

In the light of the survey results, both farmers and inhabitants of rural areas in Poland characterise a relatively high share of low physically active, according to WHO guidelines: 66% of farmers failed to exercise the minimal pro-health effort, while 45% declared literal inactivity (Table 2). For the inhabitants of rural areas, the respective shares were 49% and 32%. Observed variations are statistically significant for all typical significance levels.

**Figure 1:**
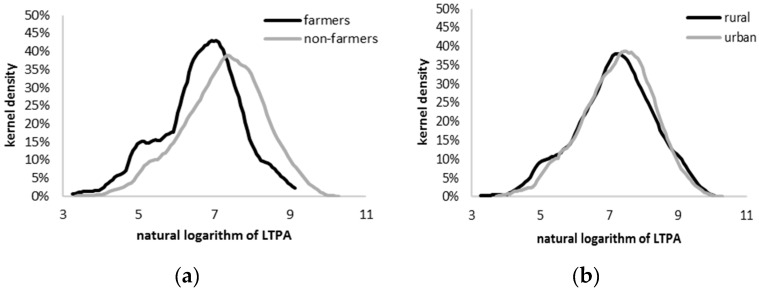
Distribution of physical activity: farming vs. others (**a**) and inhabitants of rural areas vs. others (**b**). Notes: (1) x-axis: natural logarithm of physical activity measured in MET-min; (2) For the sake of clarity and to improve fit we present kernel densities for individuals with non-zero LTPA.

The distribution of physical activity reveals striking differences between farmers and other professions. The distribution and all its statistical characteristics are shifted to the left, indicating a generally lower level of physical activity (Figure 1). To some extent this is a result of the higher frequency of total inactivity (0 MET-min/week reported) but truncating the sample to those with non-zero physical activity does not change this picture. Within that subsample, the average amount of physical activity a farmer enjoys is 788 MET-min/week, compared to 1404 among non-farmers (1220 and 1440 for rural and urban area inhabitants, respectively), and the median amount is 793 for farmers and 1486 MET-min/week for non-farmers (1365 and 1563 MET-min/week for rural and urban area inhabitants, respectively). This conclusion is supported by statistical tests, e.g., Kolmogorov-Smirnov test, confirming that the distribution of physical activity among farmers is significantly different than that among non-farmers. Similar conclusions can be drawn when inhabitants of urban and rural areas are compared. The difference narrows somewhat, but is still statistically significant.

A priori it is not clear, whether the observed gaps result from underlying differences in socioeconomic characteristics or are a genuine effect of belonging to the farming profession (or being a rural inhabitant). In order to assess the relative weight of these factors in determining the level of physical activity, we calculated several regressions. The list of statistically significant determinants of the level of LTPA consists of gender, age group, level of education, household size, region of residence, and being a farmer (for inhabitants of rural areas) or living in a rural area (for farmers) (Table 3 and Table 4). This list, however, does not overlap with the list of factors influencing the transition of individuals from being completely passive (0 MET-min/week) to active. The latter includes SES, age, household size, region of residence, frequency of internet use and, in one instance, level of education.

In general, the older the person is, the less physical activity he/she enjoys. However, the relationship is, neither linear, nor monotonic. The youngest respondents (below the age of thirty, the reference category) are clearly characterized by the highest level of physical activity. Compared to the reference group, respondents aged 30–39 years on average perform 16%–19% less, while the oldest respondents (60+ years) 36%–54% less physical activity than the youngest group. If one considers the impact of age on the odds of becoming active at all (from the selection equation), the overall pattern remains similar, with all age groups enjoying smaller odds of being active than the youngest group. The only difference here is that the relationship is not monotonic and the oldest respondents are actually more likely, on average, to be active, than middle-aged ones.

Household size is correlated negatively with the level of physical activity. For example, a respondent from a household of two people performs on average 14%–16% less activity than a single-person household (the reference category). Consistent across model specifications is the fact that this correlation holds only up to a point: respondents from 4-person households report 24%–31% less activity than single-person households, but members of even larger households enjoy only 17%–24% less physical activity. On average, females are 6%–7% less active than males, but it must be noted that the odds of being active at all is actually higher for females than males.

As far as the level of education is concerned, respondents with vocational, secondary or tertiary education have, on average, reported performing 14%–26% less physical activity than those with primary education only. Furthermore, tertiary education graduates fell to the upper side of this range, indicating that their levels of physical activity are, on average, higher than for those with vocational and secondary education. Finally, some independent variables are correlated with being active at all, but not with the level of physical activity per se. These are: Frequency of internet use and socioeconomic class (positively associated with the odds of being active).

Last but not least, being a farmer and living in a rural area matters for physical activity, but the case in not as clear-cut as it is for other socioeconomic and demographic factors. Being a farmer itself is not a statistically significant predictor of the level of physical activity, but it greatly influences being active itself. On average, the odds of being active are 46% smaller for farmers compared to non-farmers. However, once a respondent becomes active at all, the difference between farmers and non-farmers disappears. Moreover, the impact of being a farmer is only statistically significant if the type of place of residence (urban vs. rural) is controlled for. In the simplest setting (when being an inhabitant of a rural area is a statistically significant predictor of being active), farmers enjoy 26% less physical activity than non-farmers.

Finally, to account for the interplay between profession and place of residence more formally, we decided to investigate how combinations of these two variables influence both the level of physical activity and being active at all (Table 5). Precision issues notwithstanding (The number of urban-area farmers is less than 1% of the sample size) statistically significant interactions are observed. When selection into being active is considered, both being a non-farmer living in a rural area and being a farmer in a rural area are associated with lower odds of being physically active at all, compared to the control group of urban-dwelling non-farmers. Once this threshold is passed, being a farmer is the only statistically significant factor. Farmers living in urban areas are, on average, 63% less active than non-farmers, but the small sample size should be a cause for caution.

### 3.2. Sources of Variation in Physical Effort

In the previous section, we found that, on average, farmers are significantly less physically active than their counterparts performing other occupations, and inhabitants of rural areas than their urban counterparts. Age, region of residence, household size and other demographic and socioeconomic variables are also important in determining the level of physical activity—and it has also been established that farmers and non-farmers, as well as inhabitants of rural and urban areas, differ in that respect, too. Thus, we used PSM technique to calculate the difference in the amount of physical activity controlling for those factors. When considering the total reported amount of physical activity, the mean weekly shortfall across matched pairs is 730 MET-min for farmers (Cohen’s *d* = 3.09) and 249 MET-min for rural area dwellers (*d* = 2.43).

**Figure 2:**
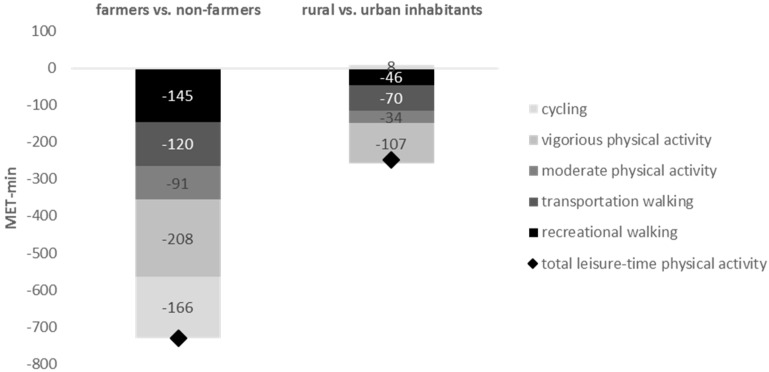
Decomposition of the total physical activity gap by types of activity.

The difference is unevenly distributed between the types of physical activity, but the general pattern holds for almost all of them. The three largest components of the gap between farmers and non-farmers are: the lack of VPA (208 MET-min/week; *d* = 5.65), cycling and recreational walking among farmers (166 and 145 MET-min/week; *d* = 5.67 and 1.93, respectively). The remaining difference is attributed to walking for transportation and MPA (120 and 91 MET-min, *d* = 0.70 and 1.82, respectively)—neither of which, however, is statistically significant. When analyzing rural and urban inhabitants, the overall physical activity gap is smaller (249 MET-min/week in favor of urban dwellers, *d* = 2.43), but its decomposition differs from the case of farmers and non-farmers. A smaller amount of walking for transportation among inhabitants of rural areas has a disproportionate impact on the total shortfall of PA—the weekly average difference in activity of this category is 107 MET-min (*d* = 2.60). This comprises 43% of total shortfall, compared to 28% in the case of farmers and non-farmers. Unfortunately, this is also the only category of PA for which the difference between inhabitants of rural and urban areas is statistically significant. Differences in all other categories, apart from statistical insignificance, are also small and do not exceed 70 MET-min/week (*d* = 1.62). Figure 2 summarizes our findings.

## 4. Discussion

This article analyses the interdependencies between LTPA and living in a rural area or being a farmer in Poland. Both groups face a challenge of physical inactivity: 66% of farmers and 49% of inhabitants of rural areas do not meet the WHO weekly amount of physical effort necessary to maintain health. Approximately two thirds of them have no activity at all—either typical sports activities, cycling or walking (In this context, it is worth mentioning that LTPA varies significantly from occupational PA, and substituting former with the latter does not bring health benefits [20]). These numbers are striking even when one takes into account the overall low activity in Poland [27] and the fact that the LTPA gap between rural and urban dwellers is common globally due to an uneven distribution of risks [28]. Barriers to LTPA for these social groups include low availability of sports facilities due to their scarcity in rural areas and the significant distance to urban facilities, lower economic status and working arrangements [29]. There is no reason to believe that any of these barriers will be eradicated in the foreseeable future, but further inefficient intervention will result in huge costs and untapped potential.

Even though Polish rural areas are strongly affected by farming, farmers and non-farmers vary significantly in terms of LTPA (For the sake of clarity, we ignore here the urban-dwelling farmers). As we have established, the mean shortfall in the amount of physical activity performed by farmers (as compared to non-farmers of most alike socioeconomic characteristics) is 730 MET-min, while rural area dwellers’ performance is almost three times lower, a mere 249 MET-min per week less compared to inhabitants of rural areas. We also found an internal variation in these shortfalls. While farmers enjoy less VPA, cycling and recreational walking than their non-farming counterparts, the difference between dwellers in rural and urban areas is primarily attributable to walking for transportation. It must be noted, however, that these Figures largely control for the differences in socioeconomic covariates and thus understate the shortfall observed in the general population. This is due to the fact that the socioeconomic make-up of farmers and (to a smaller extent) dwellers in rural areas is unfavorable for physical activity (The PSM procedure matches farmers and inhabitants of rural areas (treatment group) with non-farmers and inhabitants of urban areas, respectively (control group). The matching procedure ensures that comparison and calculation of LTPA gaps is performed across pairs of most similar socioeconomic characteristics. As a result, most respondents from the control group are not chosen at all and some are chosen more than once. Consequently, non-farmers and urban area inhabitants whose socioeconomic features differ from farmers and rural inhabitants the most are omitted from this calculation. Given the fact that their profile is likely to be LTPA-positive (i.e., above all are younger than the treatment group), this omission is understating the difference between the two groups. When different socioeconomic make-up is included, the difference in LTPA increases).

In agreement with [30,31], we have found that the amount of physical activity is negatively correlated with age—it is thus of little surprise that farmers, who are generally significantly older than other dwellers in rural areas (actually slightly younger than their urban counterparts) and the general population, are also significantly less active. There is some evidence [32] that this relationship is not linear and that physical activity tends to increase somewhat for the eldest respondents, compared to middle-aged ones, and our results confirm this as the odds of taking up any activity are higher for the 60+ group than for people aged 50–59. Due to the small sample size we are unable to confirm the same effect for farmers only. The increase in physical activity for the eldest respondents could be a result of survivorship bias. The well-established relationships between lack of PA and various non-communicable diseases suggest that the low physically active tend to have lower life expectancy in middle age. Consequently, persons exhibiting the lowest physical activity are underrepresented among the eldest, because a disproportionate number of them died before reaching 60 or older. Unfortunately, we are unable to test this hypothesis because of the cross-sectional nature of our dataset. We also find that farmers’ households tend to be smaller than those of non-farmers (and are overwhelmingly two-person households), and especially of rural area inhabitants, but that appears to be largely a by-product of higher age. While the impact of household size on physical activity is negative, for farmers this appears to be smaller than the impact of age.

Moreover, women are materially overrepresented among farmers (We hypothesize that this is mostly due to survivorship bias as there is a sizeable gap in life expectancy between women in men in Poland (at 60 the difference is 5.2 years, as compared to the EU average of 4 years). which, on balance, tends to reduce the amount of physical activity for the group. Our results here are consistent with the literature [33,34], yet we find that the relationship between gender and physical activity is non-trivial. While women are, ceteris paribus, 6%–7% less active than men, the odds of being active at all are actually higher for women than for men. A growing body of literature suggests that there is a cultural component to women’s physical activity, related to self-esteem and body ideals [35,36]. Finally, contrary to other authors’ findings, we establish that education is an LTPA-negative factor. In particular, we find that respondents with vocational, tertiary or secondary education exhibit, ceteris paribus, 14%–26% less physical activity than their counterparts who have completed only primary education. The literature suggests that the opposite should be true, as higher education is associated with more awareness of a healthy lifestyle, better planning and more control over exposure to factors decreasing physical and psychological well-being [37,38]. This puzzling result merits further investigation, which, unfortunately, lies beyond the scope of this study.

When analyzing physical activity among dwellers in rural areas in Poland, one has to take into consideration the socioeconomic circumstances in which the patterns of physical activity of this group will evolve. In the coming years, physical activity of inhabitants of rural areas will be impacted by demographic factors and structural change, and not all of these factors will work in the same direction. Firstly, this group, just like Poland’s general population, will be subject to advanced population ageing. According to the CSO’s demographic projections [39], the share of working-age residents in rural population will decrease from today’s 70% to 65% in 2030 and to 57% in 2050. On balance, this demographic transition should decrease the overall level of physical activity among dwellers in rural areas.

Secondly, the share of agriculture in total employment stands today at 11% compared to the EU average of 4% (In other words, for every five farmers in the EU, one lives in Poland), according to LFS data. This is reflected in very poor productivity Figures and production structure. Polish agriculture is among the least productive sectors (as compared to EU average) of the economy and is largely based on small-size farms. According to Eurostat data [40], 28% of arable land is fragmented into 1.1 million farms smaller than 10 ha—A type of farm virtually non-existent in France or Germany. Furthermore, the share of the largest establishments (100 ha or greater) is a mere 21% and is among the lowest in Europe. Such a structure of farming requires disproportionately high labor input (often from dependent family members) and cannot generate income sufficiently exceeding sustenance levels.

Barring institutional changes, the disproportionate employment in agriculture will decline in the years to come, as Poland is set to follow the pattern observed in virtually every developed economy [41]. The accompanying need to raise productivity in agriculture will probably result in material overhaul of the current production structure, i.e., an increase in the average size of a farm and additional investment in fixed assets. Since such structural, sectoral shifts are primarily demographic in nature [42,43] (as those employed in declining sectors are allowed to retire and are not fully replaced by new entrants), farmers as an occupational group will also age significantly. On the other hand, the tendency to increase the share of non-farming related jobs among dwellers in rural areas will, ceteris paribus, increase their aggregate physical activity. As we documented, farmers are generally less physically active than members of other professions, even when controlling for socio-economic covariates. The abovementioned structural changes are, to some extent, already occurring. According to CSO’s agricultural census [44], the average size of a farm has grown considerably, as has the share of large farms (above 30 ha). Greater emphasis on mechanization reduces the need for manual labor and, as a result, reduces occupational physical activity of farmers.

Polish rural areas are subject to social and economic changes which are natural and can be seen as part of a more complex trend of economic development—and as such should not be hindered. One should be aware, however, that various material side-effects to physical activity could happen. Minimizing them is a rationale for public intervention, which has been postulated in the literature for some time now [45]. It is bound to be a serious challenge, especially among farmers, who find it difficult even to take the first step. Perhaps their lifestyle (attitudes, needs, awareness, norms) is to blame, but this view is far from being a consensus one [29,46]. Perhaps this attitude stems from insufficient health education. Nowadays, health education in rural environments faces many hurdles associated with under-appreciation of prophylaxis, lack of necessary infrastructure, insufficient activity on the part of local governments and a lack of established role models of pro-health behavior and skilled health promoters [45]. Nevertheless, sudden changes are unlikely and public intervention should be narrowly targeted and should focus on activities different from regularly performed physical work, simple in form and characterized by low transaction costs or compensating for side-effects of physical work on a farm e.g. back pain [47].

This article casts new light on LTPA in Polish rural areas. The utilized dataset covers a whole country (a CEE country, rare itself). Furthermore, we select farmers from inhabitants of rural areas, which is even less common (with the noteworthy exception of [48]). Despite these advantages, we are fully aware of the shortcomings of the dataset. As it represents a cross-sectional dimension, no dynamic conclusions can be drawn. Moreover, we have to rely on physical activity declarations. Finally, as working time in farming varies significantly through the year, it would be desirable to test for potential variations in the annual cycle. We are also aware of the fact that the category of rural area inhabitants or farmers may be defined differently and these definitions affect the final results.

## 5. Conclusions

Rural areas in Poland are in a precarious situation—their traditional socioeconomic model, based on labor-intensive and small-scale agriculture, is likely to be unsustainable. The required adjustment will have a profound impact on, among other things, the well-being and health of the residents of rural areas. From the perspective of health, the increasing diversification of economic activity will be a positive development per se, since it will reduce the number of individuals most at risk through insufficient physical activity and its side-effects. On the other hand, farmers as a group must be targeted by public intervention aiming to increase their awareness of the advantages of maintaining LTPA. This is not only because of the fact that farming itself is a negative factor for physical activity, but also due to the increased mechanization of agriculture, which will reduce the amount of manual labor and depress the aggregate physical activity of future farmers. Finally, the effect of ageing is definitely negative on physical activity—the older the person, the smaller the amount of LTPA performed. However, we believe that there is a silver lining to ageing. Since it is impossible to distinguish cohort effects from the impact of age, it is possible that as current young farmers age, the declines in physical activity experienced by them over time will be smaller than the current cross-section of the population suggests.

## Figures and Tables

**Table 1:** Sample and subsamples.

Variables		Rural	Urban	Farmers	Non-Farmers	Total
gender	male	552	925	92	1385	1477
	female	620	954	141	1433	1574
age (year)	15–29	280	406	6	680	686
	30–39	236	348	25	559	584
	40–49	171	284	27	428	455
	50–59	174	336	47	463	510
	60+	311	505	128	688	816
household size (persons)	1	136	352	44	444	488
	2	337	647	95	889	984
	3	272	453	29	696	725
	4	226	306	24	508	532
	5+	201	121	41	281	322
education	primary	289	247	107	429	536
	vocational	423	568	98	893	991
	secondary	357	730	24	1063	1087
	tertiary	103	334	4	433	437
profession	executives, owners	36	120	0	156	156
	professionals	67	217	0	284	284
	other white-collar workers	52	168	0	220	220
	qualified blue-collar workers	302	473	0	775	775
	non-qualified blue-collar workers	45	92	0	137	137
	farmers	210	23	233	0	233
	homemakers	49	41	0	90	90
	pupils, students	95	121	0	216	216
	pensioners	221	490	0	711	711
	unemployed	95	134	0	229	229
place of residence	rural areas	1172	0	210	962	1172
	urban areas, <50 K inhabitants	0	702	14	688	702
	urban areas, 50 K–500 K inhabitants	0	836	9	827	836
	urban areas, >500 K inhabitants	0	341	0	341	341
region	central	216	397	52	561	613
	northeastern	180	215	65	330	395
	northwestern	272	460	44	688	732
	southwestern	216	537	22	731	753
	southeastern	288	270	50	508	558
wave	1	389	627	76	940	1016
	2	396	624	71	949	1020
	3	387	628	86	929	1015
TOTAL			1172	1879	233	3051

**Table 2:** Prevalence of low physical activity and physical inactivity.

Variable		Low Physically Active (0–600 MET-min/week)	*p*-Value	Physically Inactive (0 MET-min/week)	*p*-Value
overall		41.1%		24.0%	
place of residence	rural areas	49.3%	<0.01	31.7%	<0.01
	urban areas	35.7%		18.3%	
profession	farming	65.6%	<0.01	44.5%	<0.01
	non-farming	39.1%		21.6%	

**Table 3:** Impact of farming on physical activity—estimates of Heckman selection model.

Model	Variable		Coefficient	Adjusted Coefficient	Standard Error	*p*-Value	95% Confidence Interval	
outcome equation	gender	male	ref.					
		female	−0.098	−0.065	0.049	0.048	−0.194	−0.001
	household size (persons)	1	ref.					
		2	−0.114	−0.158	0.077	0.137	−0.265	0.036
		3	−0.175	−0.265	0.087	0.044	−0.344	−0.005
		4	−0.254	−0.306	0.089	0.005	−0.429	−0.078
		5+	−0.131	−0.245	0.102	0.201	−0.332	0.070
	age group (year)	15–29	ref.					
		30–39	−0.020	−0.188	0.078	0.794	−0.172	0.132
		40–49	−0.112	−0.293	0.080	0.164	−0.269	0.046
		50–59	−0.267	−0.416	0.088	0.002	−0.439	−0.094
		60+	−0.453	−0.540	0.080	<0.001	−0.610	−0.297
	education	primary	ref.					
		vocational	−0.199		0.072	0.006	−0.339	−0.058
		secondary	−0.170		0.069	0.013	−0.305	−0.036
		tertiary	−0.162		0.081	0.045	−0.320	−0.004
	region	central	ref.					
		northeastern	0.126	0.024	0.088	0.153	−0.047	0.300
		northwestern	0.200	0.152	0.073	0.006	0.057	0.343
		southwestern	0.289	0.192	0.071	<0.001	0.501	0.427
		southeastern	−0.001	−0.038	0.077	0.900	−0.160	0.140
	const		8.002		0.108	<0.001	7.791	8.214
selection equation	gender	male	ref.					
		female	0.70		0.050	0.160	−0.028	0.167
	household size (persons)	1	ref.					
		2	−0.093		0.078	0.228	−0.245	0.058
		3	−0.194		0.089	0.028	−0.367	−0.021
		4	−0.112		0.095	0.237	−0.298	0.074
		5+	−0.244		0.102	0.017	−0.444	−0.044
	age group (year)	15–29	ref.					
		30–39	−0.361		0.082	<0.001	−0.521	−0.201
		40–49	−0.389		0.086	<0.001	−0.556	−0.221
		50–59	−0.320		0.091	0.001	−0.500	−0.141
		60+	−0.188		0.090	0.038	−0.364	−0.011
	socioeconomic class	upper middle class	ref.					
		middle class	−0.152		0.096	0.115	−0.340	0.037
		lower middle class and skilled working class	−0.2496		0.0913	0.006	−0.429	−0.071
		working class	−0.2974		0.1023	0.004	−0.498	−0.097
		not working	−0.4143		0.1118	<0.001	−0.6334	−0.195
	internet use	everyday	ref.					
		rarely	−0.1396		0.0592	0.018	−0.256	−0.024
		never	−0.3250		0.0665	<0.001	−0.4553	−0.1947
	profession	non-farmer	ref.					
		farmer	−0.464		0.083	<0.001	−0.627	−0.300
	region	central	ref.					
		northeastern	−0.220		0.088	0.013	−0.393	−0.047
		northwestern	−0.104		0.078	0.180	−0.256	0.048
		southwestern	−0.208		0.075	0.006	−0.355	−0.060
		southeastern	−0.060		0.082	0.464	−0.221	0.101
	const		1.630		0.141	<0.001	1.354	1.906
	*atanh (ρ)*		−1.271		0.120	<0.001	−1.505	−1.037
	ln (σ)		0.221		0.025	<0.001	0.172	0.271
	*ρ*		−0.854		0.032		−0.906	−0.777
	*σ*		1.248		0.032		1.188	1.311
	*λ*		−1.066		0.064		−1.192	−0.940

Notes: (1) Ref.—reference category. (2) Heckman selection model was jointly estimated using maximum likelihood method. (3) Coefficients for independent variables present both in the outcome and in the selection equation were adjusted to allow for direct interpretation, using the method established by [26]). (4) Dependent variable is the natural logarithm of the weekly amount of physical activity increased by 1. (5) *ρ* is the correlation of residuals from the outcome and selection equations, its inverse hyperbolic tangent, atanh(ρ), is used to establish statistical significance of *ρ*. (6) σ is the standard error of residuals from the outcome of equation. (7) λ=ρσ.

**Table 4:** Impact of living in a rural area on physical activity—estimates of Heckman selection model.

Model	Variable		Coefficient	Adjusted Coefficient	Standard Error	*p*-Value	95% Confidence Interval	
outcome equation	gender	male	ref.					
		female	−0.089	−0.060	0.049	0.068	−0.185	0.007
	household size (persons)	1	ref.					
		2	−0.136		0.067	0.043	−0.267	−0.005
		3	−0.224		0.076	0.003	−0.373	−0.075
		4	−0.243		0.078	0.002	−0.396	−0.090
		5+	−0.167		0.089	0.060	−0.340	0.007
	education	primary	ref.					
		vocational	−0.255	−0.194	0.080	0.001	−0.412	−0.098
		secondary	−0.189	−0.129	0.075	0.012	−0.337	−0.042
		tertiary	−0.210	−0.084	0.089	0.019	−0.385	−0.034
	profession	non-farmer	ref.					
		farmer	−0.255		0.106	0.016	−0.464	−0.047
	region	central	ref.					
		NE	0.130	0.023	0.088	0.141	−0.043	0.302
		NW	0.190	0.149	0.072	0.008	0.050	0.331
		SW	0.265	0.180	0.070	<0.001	0.129	0.402
		SE	−0.017	−0.042	0.075	0.822	−0.164	0.130
	const		8.039		0.107	<0.001	7.830	8.249
selection equation	gender	male	ref.					
		female	0.069		0.051	0.183	−0.032	0.169
	age group (year)	15–29	ref.					
		30–39	−0.406		0.086	<0.001	−0.574	−0.237
		40–49	−0.431		0.090	<0.001	−0.607	−0.256
		50–59	−0.386		0.094	<0.0001	−0.569	−0.202
		60+	−0.239		0.087	0.006	−0.410	−0.069
	education	primary	ref.					
		vocational	0.142		0.076	0.061	−0.007	0.291
		secondary	0.138		0.076	0.067	−0.010	0.286
		tertiary	0.291		0.097	0.003	0.101	0.482
	internet use	everyday	ref.					
		rarely	−0.150		0.063	0.016	−0.273	−0.028
		never	−0.344		0.071	<0.001	−0.482	−0.205
	place of residence	urban areas	ref.					
		rural areas	−0.285		0.055	<0.001	−0.392	−0.177
	region	central	ref.					
		northeastern	−0.248		0.090	0.006	−0.424	−0.073
		northwestern	−0.096		0.079	0.227	−0.251	0.059
		southwestern	−0.199		0.076	0.009	−0.347	−0.050
		southeastern	−0.059		0.082	0.474	−0.220	0.102
	const		1.256		0.100	<0.001	1.059	1.452
	*atanh (ρ)*			−1.134		0.153	<0.001	−1.433
	*ln (σ)*			0.197		0.032	<0.001	0.135
	*ρ*			−0.813		0.052		−0.892
	*σ*			1.218		0.039		1.145
	*λ*			−0.989		0.092		−1.170

Notes: (1) ref—reference category. (2) Heckman selection model was jointly estimated using maximum likelihood method. (3) Coefficients for independent variables present both in the outcome and in the selection equation were adjusted to allow for direct interpretation, using the method established by [26]. (4) Dependent variable is the natural logarithm of the weekly amount of physical activity increased by 1. (5) *ρ* is the correlation of residuals from the outcome and selection equations, its inverse hyperbolic tangent, atanh (ρ), is used to establish statistical significance of *ρ*. (6) σ is the standard error of residuals from the outcome equation. (7) λ=ρσ.

**Table 5:** Impact of living in a rural area and farming on physical activity—estimates of Heckman selection model.

Model	Variable		Coefficient	Adjusted Coefficient	Standard Error	*p*-Value	95% Confidence Interval	
outcome equation	gender	male	ref.					
		female	−0.096	−0.062	0.049	0.051	−0.192	0.001
	household size (persons)	1	ref.					
		2	−0.153		0.067	0.022	−0.285	−0.021
		3	−0.256		0.076	0.001	−0.405	−0.106
		4	−0.289		0.079	<0.001	−0.443	−0.135
		5+	−0.223		0.090	0.014	−0.400	−0.046
	age group (year)	15–29	ref.					
		30–39	−0.034	−0.163	0.077	0.660	−0.186	0.118
		40–49	−0.123	−0.212	0.080	0.123	−0.280	0.033
		50–59	−0.280	−0.275	0.087	0.001	−0.450	−0.110
		60+	−0.476	−0.362	0.078	<0.00	−0.628	−0.324
	education	primary	ref.					
		vocational	−0.198		0.072	0.006	−0.339	−0.057
		secondary	−0.160		0.069	0.020	−0.294	−0.025
		tertiary	−0.141		0.081	0.081	−0.299	0.017
	region	central	ref.					
		northeastern	0.123	0.028	0.088	0.162	−0.049	0.295
		northwestern	0.201	0.154	0.072	0.006	0.059	0.343
		southwestern	0.282	0.186	0.071	<0.001	0.144	0.420
		southeastern	−0.019	−0.041	0.076	0.799	−0.168	0.130
	professional status *×* place of residence	non-farmer, urban area	ref.					
		non-farmer, rural area	0.047	−0.065	0.058	0.414	−0.067	0.160
		farmer, urban area	−0.720	−0.633	0.308	0.019	−1.324	−0.117
		farmer, rural area	0.071	−0.220	0.135	0.601	−0.194	0.336
	const		8.028		0.106	<0.001	7.821	8.235
selection equation	gender	male	ref.					
		female	0.076		0.050	0.134	−0.023	0.174
	age group (year)	15–29	ref.					
		30–39	−0.365		0.083	<0.001	−0.527	−0.202
		40–49	−0.397		0.087	<0.001	−0.568	−0.226
		50–59	−0.338		0.091	<0.001	−0.516	−0.161
		60+	−0.184		0.086	0.033	−0.352	−0.015
	socioeconomic class	upper middle class	ref.					
		middle class	−0.151		0.010	0.129	−0.346	0.044
		lower middle class and skilled working class	−0.227		0.095	0.017	−0.412	−0.041
		working class	−0.269		0.106	0.011	−0.476	−0.061
		not working	−0.346		0.113	0.002	−0.568	−0.124
	internet use	everyday	ref.					
		rarely	−0.126		0.062	0.040	−0.247	−0.006
		never	−0.289		0.069	<0.001	−0.424	−0.154
	region	central	ref.					
		northeastern	−0.212		0.089	0.017	−0.387	−0.037
		northwestern	−0.105		0.079	0.182	−0.259	0.049
		southwestern	−0.214		0.076	0.005	−0.362	−0.065
		southeastern	−0.049		0.082	0.554	−0.210	0.112
	professional status *×* place of residence	non-farmer, urban area	ref.					
		non-farmer, rural area	−0.251		0.057	<0.001	−0.363	−0.139
		farmer, urban area	0.195		0.335	0.561	−0.462	0.852
		farmer, rural area	−0.649		0.101	<0.001	−0.847	−0.450
	const		1.559		0.122	<0.001	1.319	1.798
	*atanh (ρ)*		−1.203		0.135	<0.001	−1.468	−0.938
	*ln (σ)*		0.208		0.028	<0.001	0.153	0.263
	*Ρ*		−0.835		0.041		−0.899	−0.734
	*Σ*		1.231		0.035		1.165	1.301
	*Λ*		−1.027		0.077		−1.178	−0.877

Notes: (1) ref.—reference category. (2) Heckman selection model was jointly estimated using maximum likelihood method. (3) Coefficients for independent variables present both in the outcome and in the selection equation were adjusted to allow for direct interpretation, using the method established by [26]. (4) Dependent variable is the natural logarithm of the weekly amount of physical activity increased by 1. (5) *ρ* is the correlation of residuals from the outcome and selection equations, its inverse hyperbolic tangent, atanh (ρ),  is used to establish statistical significance of *ρ*. (6) σ is the standard error of residuals from the outcome equation. (7) λ=ρσ.

## Abbreviations

CEE	Central and Eastern Europe
CSO	Central Statistical Office of Poland
IPAQ	International Physical Activity Questionnaire
LTPA	Leisure-Time Physical Activity
MPA	Moderate Physical Activity
NCDs	Noncommunicable Diseases
PA	Physical Activity
PSM	Propensity Score Matching
SES	Socioeconomic Status
VPA	Vigorous Physical Activity
WHO	World Health Organization
